# Rearing Sea Urchins to Promote ‘Ready-to-Spawn’ Conditions for Ecotoxicological Surveys

**DOI:** 10.3390/toxics13080705

**Published:** 2025-08-21

**Authors:** Roberta Miroglio, Pietro Soro, Lisa Zanetti, Laura Castellano, Natalia Perez, Erica Carlig, Marco Faimali, Chiara Gambardella

**Affiliations:** 1 National Research Council, Institute of the Anthropic Impact and Sustainability in the Marine Environment (CNR-IAS), Via de Marini 16, 16149 Genova, Italy; roberta.miroglio@ias.cnr.it (R.M.); lisa.zanetti@ias.cnr.it (L.Z.); erica.carlig@ias.cnr.it (E.C.); marco.faimali@cnr.it (M.F.); 2 Costa Edutainment SpA-Acquario di Genova, Area Porto Antico, Ponte Spinola, 16128 Genova, Italy; lcastellano@costaedutainment.com (L.C.);

**Keywords:** bioassay, ecotoxicology, embryotoxicity, fertilization, sea urchins

## Abstract

The sea urchin *Paracentrotus lividus* is a good model in ecotoxicology, but adults living along the Italian coasts have a limited reproductive period. In this species, natural or human-driven pressures may lead to limited gamete availability for ecotoxicological surveys. This study investigates the quality of early developmental stages of wild and cultured sea urchins to be used in ecotoxicology, avoiding field collection of mature specimens. Adult sea urchins were cultured in the laboratory for 2 years. Every 45 days, fertilization and larval quality were checked and compared to those from adults sampled in the wild. Fertilization was never affected, differently from development, which was impaired in the larvae obtained from sea urchins reared for more than one year. Fertilization and embryotoxicity were performed using copper nitrate in wild and cultured sea urchins. Fertilization did not differ up to ten months, while similar embryotoxicity was only found up to 5 months. This study promotes rearing sea urchins in ‘ready-to-spawn’ conditions for ecotoxicology surveys by recommending 10- and 5-month rearing times to assess fertilization and embryo toxicity, respectively. Here, we provided a baseline in marine ecotoxicology to obtain gametes on demand, irrespective of reproductive period and other pressures that may impact gamete availability.

## 1. Introduction

The common sea urchin *Paracentrotus lividus* (Lamarck, 1816) is an edible echinoid with a wide distribution range across the Mediterranean and Northeast Atlantic. It is a keystone species in the benthic community, since its grazing activity helps regulate macrophyte populations [1]. Due to its sensitivity to contaminants, this species is currently used to assess environmental quality [2,3,4,5], and it is a model organism for ecotoxicological surveys. Gametes and embryos are necessary to perform fertilization and carry out developmental bioassays as required by national and international regulations [6,7,8]. Moreover, its sensitivity to pollutants, its well-studied and short life cycle, fast development, and other factors (i.e., fertilization in water, transparent eggs, and high offspring production) are promoting sea urchins as a model in ecotoxicology. Another important basic requirement is the species availability throughout the year [9]. Adult specimens of *P. lividus* living along the Italian coasts have a limited reproductive period from October to June. Gametogenesis occurs during the coldest months [10,11]. Moreover, natural (i.e., seasonal fluctuations) or human-driven pressures and subsequent limited gamete availability [12,13,14] may affect ecotoxicological investigations. Population dynamics of *P. lividus* in the Italian coasts are mainly affected by overfishing and sea warming rather than the pressure exerted by the removal of adults for ecotoxicological surveys [15]; however, it is important to maintain sea urchins in ‘ready-to-spawn’ conditions to obtain gametes on demand [9], avoiding recurrent field collection of mature specimens and preserving natural populations.

Several studies have been performed so far on long term maintenance in culture of the sea urchin *P. lividus*, also to meet echinoculture production needs [9,16,17,18,19,20,21,22,23]. Through gonad index and histological analyses from wild and/or hatchery-raised sea urchins, they analyzed environmental parameters and feed regimes to assess and improve gonad development. For instance, a different photoperiod (i.e., 24 h dark, 8 h light, 16 h dark, 16 h light, and 8 h dark), temperature (18–26 °C) and diet based on vegetal meals (i.e., natural algae, macroalgae, lettuce, maize, and dried seaweed) or combining vegetal with animal sources (i.e., fish, mussel, krill, and anchovy) were used in *P. lividus* echinoculture to improve gonad development [9,24,25,26,27,28,29,30].

Spawning is a non-invasive method that provides an indirect assessment of reproductive ability. Applying this method to assess sea urchin fertilization ability and early developmental stage quality from wild and cultured populations is necessary to establish ‘ready-to-spawn’ conditions for ecotoxicological purposes. Planktonic and early developmental stages (gametes, embryos, and larvae) are the most sensitive phases in the whole sea urchin life cycle [31]. Thus, these early life cycle stages have been used worldwide in several toxicity bioassays to assess and monitor water and sediment toxicity [32,33,34]. For instance, early sea urchins stages have been included in regulatory toxicology—by performing fertilization assays, development, and embryotoxicity tests—to investigate the effects of several substances and compounds (i.e., chemicals, nanomaterials, pharmaceuticals, and microplastics [2,13,14,35,36]). In addition, some bioassays have been suggested for standardization or OECD adoption [8,37,38]. According to OECD guidelines [39] that promote sea urchin bioassays, the Environmental Canada guidelines [40] include fertilization and short-term sub-chronic test methods on different sea urchin species to assess the effects of toxicants. Moreover, other scientific institutions have recommended the use of sea urchin embryos as bioassay testing models for environmental monitoring programs and regulatory toxicology [41].

The aim of this study was to assess the quality of gametes and early developmental stages of sea urchins matured in confined conditions to avoid continuous sampling of adults from the natural environment, especially during summer, and to obtain zygotes and embryos on demand for ecotoxicological studies. To achieve this goal, fertilization and embryo assays were performed on both natural and cultured sea urchin populations for two years. This rearing span was selected since, to our knowledge, no study on spawning exceeds one year [28]. Also, ecotoxicological bioassays were performed using copper as a reference toxicant [42] to compare their effective concentration (EC50) outcome on 50% of organisms in fertilized eggs and on larvae from natural (wild) sea urchins and 50% on cultured ones, as well as checking offspring quality to be used in ecotoxicological investigations. We performed both fertilization and embryo toxicity tests, currently used and recommended in standardized protocols for water and sediment quality assessment [6,7,8,28,29,30]. Due to the current lack of standardized methods in sea urchin ecotoxicology and considering that several parameters may affect echinoderm sensitivity [14,28], this study also aims at increasing knowledge on laboratory conditions to culture sea urchins to enhance reproducibility, relevance to field conditions, and animal welfare.

## 2. Materials and Methods

Two laboratory experiments were carried out. One experiment (Experiment I) was performed to verify the quality of gametes and larvae from natural and cultured sea urchins for two years. In this experiment, fertilized eggs and larval developmental percentages were checked after 40 min and 72 h, respectively [42]. In the second experiment (Experiment II), fertilization and embryotoxicity tests were performed by using early life stages of wild and cultured sea urchins exposed to copper nitrate, which is a reference toxicant for ecotoxicological use [35].

- **Wild population sampling**

Twenty individuals of *P. lividus* were collected from Ligurian coasts (Western Mediterranean, 44°23′22.92″ N latitude, 8°58′23.99″ E longitude) every 45 days from March 2021 to April 2023. Sea urchins were selected and chosen to be uniform in size (diameter > 40 mm), corresponding to adult stages [9]. They were brought to the laboratory in a refrigerated bag and used to perform the experiments. A total of eighteen samplings (1 March 2021, 16 April 2021, 28 May 2021, 9 July 2021, 27 August 2021, 15 October 2021, 30 November 2021, 14 January 2022, 1 March 2022, 15 April 2022, 30 May 2022, 14 July 2022, 31 August 2022, 13 October 2022, 30 November 2022, 12 January 2023, 28 February 2023, and 13 April 2023) were conducted along the Ligurian coasts. During summer, sea urchins were always collected in the same geographic area reported above but at deeper waters (3 m depth versus 1–1.5 m depth used in other seasons) to compensate for organism absence in shallow areas.

- **Cultured population sampling**

During the first sampling (March 2021), forty sea urchins (diameter > 40 mm) were collected to be further cultured, besides the twenty-one used in the previous paragraph. They were maintained in aquaria at the Aquarium of Genoa (Italy) for two years in a 2000 L recirculating tank with natural filtered seawater (FSW, temperature 16 ± 1 °C, salinity 35–37‰, pH 8.00–8.20, photoperiod 12 h light: 12 h dark, according to [43]), and continuously aerated. Optimal seawater parameters were maintained in the tank for three months before sea urchin sampling, thanks to a system equipped with a sand filter, a cartridge filter for the removal of nitrates and nitrites, a plastic biological filter (i.e., “bioballs”) as a substrate for nitrifying bacteria, and an ultraviolet sterilizer. The presence of a pump allowed recirculating seawater. Water changes of the tank (10% total volume) took place once a week. Optimal physical and chemical conditions of the seawater were measured daily. Temperature, salinity, dissolved oxygen, and pH were measured using a multiparametric probe (Hanna Instrument, Veneto, Italy; Supplementary Table S1). The concentrations of nitrates, nitrites, ammonium, and phosphates were measured in the tank by using commercial kits. Before starting the experiments, small rocks covered by biofilm were collected in the same area of sea urchin sampling. The rocks were placed into the tank before introducing animals to recreate a natural substrate in a laboratory setting. At the time of sampling, forty sea urchins were raised in this tank, and their health was checked daily (i.e., by observing any spots on body surfaces or partial spine loss).

During the whole experimental setup, adults were fed three times a week with a natural diet (about 5 g per individual, according to [27]), mainly based on natural raw materials as a source of proteins, carbohydrates, and lipids that promote gamete production in livestock sea urchins [9]. Specifically, the diet consisted of lettuce, carrots, corn, algae (i.e., *Ulva* sp. [44,45]), agar, fish oil, and mineral supplements (calcium carbonate; Table A1). The major ingredients were algal components (43%, Table A1) and carotenoids (i.e., carrots and corn, 22%), since they stimulate gonad growth and maturation in sea urchins [45,46,47]. All cultured animals ate almost all ingredients, without selectively eating the different components. Before each feed provision, the tank was cleaned, removing uneaten food and fecal pellets by siphoning. The sea urchins collected during the first sampling and cultured for two years were induced to spawn every 45 days to check fertilization and development up to 72 h. This reconditioning time was selected according to previous studies that recommend 21–60 days for gonad maturation in sea urchin broodstock [28,48].

- **Spawning**

Twenty adults collected in the field and cultured in the aquaria were randomly selected to be spawned by intracoelomic injection of 0.5 mL KCl (0.5 M) diluted in 0.22 filtered seawater (FSW). This number ensured a minimum of three spawning animals per sex to perform the experiments in each season [14]. Cultured sea urchins were starved for three days before inducing spawning [49] to reduce KCl injection; then, a maximum of twenty specimens out of forty were forced to spawn each time. Once three males and females were found, no other sea urchins were injected. This allowed us not to stress all cultured sea urchins every 45 days, promoting recondition for spawning in the laboratory. After injection, sea urchins were gently shaken by hand until spawning started to avoid damaging internal structures [50]. Dry sperm were collected from the genital pores by Pasteur pipette and maintained at 4 °C in aliquots of 200 µL in tubes before use [51]. Sea urchin eggs were collected in FSW at room temperature. Sperm motility and egg quality were checked before fertilization. Specifically, egg quality was checked by evaluating the presence of a visible nucleus and regular shape; immature oocytes, eggs with damaged or lacking jelly coats, and eggs with irregular shapes and sizes were not used for the experiments. Sperm motility was checked under an inverted microscope (Leica, Wetzlar, Germany), according to [35]. Females and males were let spawn for 30 min [52]. After KCl induction, cultured and wild sea urchins were placed back into the aquaria and in the field, respectively.

- **Experiment I—Quality Assessment of Early Life Stages**

To assess early life stages from wild and cultured sea urchins, fertilization tests were performed for a total of 18 samplings/times. Sperm and eggs from three different specimens were mixed, respectively. Then, approximately 1000 eggs/mL concentration was used in 6 multiwell capsules containing 10 mL FSW. Sperm density was determined by adding 25 mL of freshwater to 50 μL of sperm from three males to achieve a 500-fold dilution, following the Italian Institute for Environmental Protection and Research guidelines [42]. The number of sperm present in 10 μL suspension was determined using a hemocytometer (Thoma chamber) under an optical inverted microscope (Leica DMi1, 40× magnification). This calculation was used to determine how much sperm to add to eggs to obtain a sperm-egg ratio of 15,000:1 for both fertilization and larval development tests [42,53]. Regarding the fertilization test, 0.1 mL of sperm suspension of wild and cultured sea urchins was incubated with 8.9 mL of FSW in the dark for one hour at 18 ± 1 °C in multiwall capsules; then, 1 mL of eggs was added to check the fertilization rate [42]. The experiment was carried out in six replicates. After 40 min, samples were fixed in 2% paraformaldehyde (PAF), and fertilization success was checked under a stereomicroscope (Discovery V.8, Zeiss, Jena, Germany) with 8× magnification by assessing fertilization membrane development. To check larval development, the fertilized eggs from wild and cultured sea urchins were left to develop for 72 h to reach the 4-armed pluteus (larval) stage in dark conditions at 18 ± 1 °C, by performing six replicates. Then, the sea urchins were fixed in 2% PAF, and the development was checked under a stereomicroscope (Discovery V.8, Zeiss, Germany) under 8× magnification to determine the percentage of anomalies, including any delayed and anomalous larvae [5]. The latter consisted of skeletal anomalies, such as truncated plutei, asymmetrical or bent larval bodies, incomplete or absent arms, or crossed-tip skeletal rods. The acceptability of sea urchin test results to be further used in toxicity tests (Experiment II) was set when both fertilization and normal development percentages are >80%. This was in line with previous ecotoxicological studies where sea urchin unfertilized egg rate or anomalous development in control samples did not exceed 20% [42,54,55].

- **Experiment II—Toxicity Tests**

The same above-described experimental setup was applied in Experiment II, where gametes (sperm) and fertilized eggs were exposed to copper nitrate. A solution of copper nitrate (Cu(NO_3_)_2_·3H_2_O; Merck, Milan, Italy) of 10 mg/L was prepared in distilled water from a stock solution (1000 g/L). Then, increasing nominal concentrations of copper nitrate (0-12-24-36-48-60-72-84-96 µg/L) were prepared in FSW and used to assess fertilization and embryotoxicity tests. This experiment aimed to estimate the dose-response curve and verify the potential differences in fertilization rate and larval development in the early life stages of sea urchins in either natural (wild) or cultured conditions. The fertilization test (spermiotoxicity test) was performed according to the methodology described in the previous section by exposing sperm suspension of wild and cultured sea urchins to copper nitrate for one hour and then by adding eggs. After 40 min, fertilization success was assessed. Regarding embryotoxicity, fertilized eggs from wild and cultured sea urchins were exposed to copper nitrate and left to develop in the dark for 72 h to evaluate 4-armed pluteus stage development.

Six replicates were carried out for each test. After exposure, sea urchins were fixed in 2% PAF. Fertilization and development percentages were checked under a stereomicroscope (Discovery V.8, Zeiss, Germany, 8× magnification) as reported above. ‘Non-fertilized eggs’ and ‘abnormal larvae’ rates were used to establish EC50. ‘Abnormal larvae’ were referred to as larvae showing asynchronous or delayed development, morphological asymmetry, supernumerary rods, and deformed/absent arms [5].

- **Statistical Analyses**

All data are expressed as means ± standard error of the six replicates. For statistical analyses, data were grouped in four-month periods (Table A2). For Experiment II, the effective concentration (i.e., EC50, namely copper nitrate concentration resulting in 50% effect in the exposed sea urchin gametes and larvae after 40 min and 72 h, respectively) and related 95% confidence limits (C.L.) were calculated using Trimmed Spearman Karber (TSK) analysis. In Experiment II, responses to each treatment (fertilized eggs percentage, percentage of normal larval development) in control tests were corrected for effects by applying the Abbott’s formula, according to [50]. Significant differences among samples (fertilized eggs, larvae) were determined using two-way analysis of variance (ANOVA) considering the ‘treatment’ (wild vs. cultured sea urchins) factor and the ‘period’ (P) factor. The latter included one semester, corresponding to four spawning events. By comparing these periods, it was possible to determine significant differences by using the same number of spawning events.

When data failed to meet the assumption of normality or homogeneity of variances, permutational ANOVA (PERMANOVA) was carried out. This analysis is based on Euclidean distance; a maximum of 9999 permutations was used to obtain the *p*-values (α < 0.05) in each dataset, applying Monte Carlo correction. Data analysis was performed using R statistical software (R version 4.0.2) for ANOVA; PRIMER 6 software implemented with the Permanova + routine was used for PERMANOVA.

## 3. Results

### 3.1. Experiment I—Quality Assessment of Early Life Stages

**Figure 1:**
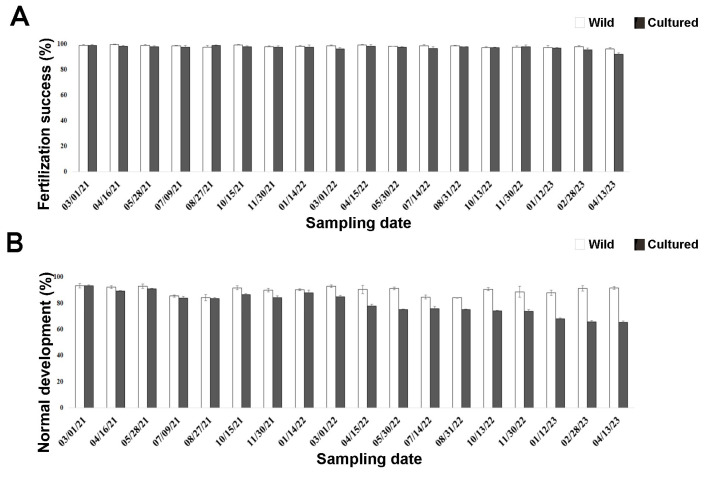
Quality assessment of early life stages in wild (white bars) and cultured (black bars) sea urchins during the two-year experiment. (**A**) Fertilization success rate after 40 min. (**B**) Larval development rate after 72 h post fertilization.

No mortality was recorded for any cultured sea urchins in the two-year rearing period. Fertilization occurred in both wild and cultured sea urchins after all sampling times (Figure 1A), ranging from 96.35% to 99.10% in wild sea urchins and from 92.04% to 98.47% in cultured sea urchins. However, a high number of adults sampled in the wild were induced to spawn during summer to assess gamete quality compared to cultured sea urchins. No significant interaction between treatment and periods (*p* > 0.05) was observed.

Larval development occurred in sea urchins after all sampling times (Figure 1B). However, significant differences (*p* = 0.0006) were observed between the two treatments from April 2022 (third period) onwards (Table A3). Larvae from wild sea urchins showed a high percentage of normal development (84.20–93.33%; Figure 1B) that did not differ among sampling times (*p* > 0.05). Similar results were observed for cultured sea urchins up to March 2022 (first–second period), when 85% of them developed normally. However, this percentage significantly decreased with time in the aquaria (Table A4), reaching the minimum in April 2023 (65.25%).

No significant differences between wild and cultured sea urchins were observed up to August 2021 (first period), unlike the other periods. In particular, larval development was significantly higher in wild sea urchins than in cultured ones (Table A5). However, since less than 20% of the larvae from sea urchins reared in the aquaria for one year had developmental anomalies (March 2021–March 2022; Supplementary Figure S1), they could still be used for embryotoxicity tests (Experiment II).

### 3.2. Experiment II—Toxicity Tests

EC50 measured during the fertilization tests (spermiotoxicity) using copper nitrate ranged from 41.29 µg/L to 54.46 µg/L in wild sea urchins. Conversely, cultured sea urchins reported a wider EC50 range (13.99–44.25 µg/L; Table 1). Overall, similar toxicity values were observed between wild and cultured sea urchins up to January 2022, with overlapping confidence limits. After this time corresponding to 10 months of rearing, EC50 gradually decreased until April 2023. Overall, a significant effect between treatments and periods (*p* < 0.0001) was observed. EC50 was similar in wild and cultured sea urchins until August 2021 (corresponding to the first period), while it significantly differed in the other periods (*p* = 0.0470, *p* < 0.0001, and *p* < 0.0001 in the second, third, and fourth periods, respectively), being higher in the wild than in cultured sea urchins.

Embryotoxicity ranged between 37.40 and 55.24 µg/L in wild sea urchins, being the lowest in summer. EC50 observed in cultured sea urchins (17.27–40.99 µg/L) was lower than in the wild ones. Overall, EC50 and confidence limits would overlap in wild and cultured sea urchins until July 2021 (Table 2). Then, a gradual decrease in toxicity was observed since August 2021 in cultured sea urchins. The interaction between treatment and period (*p* = 0.0003) was found to be significant. Toxicity tolerance in wild and cultured sea urchins was not significantly different in the first period, unlike toxicity rates in those from longer cultivation periods. Specifically, toxicity tolerance in wild sea urchins was significantly higher than in those sea urchins cultured since April 2022 (second period; *p* = 0.0001).

## 4. Discussion

In this study we successfully checked the quality of gametes and early development stages of sea urchins matured in confined conditions for their use in ecotoxicological tests.

Considering the importance of reducing the collection of sea urchins in the field while preserving natural populations, we induced sea urchin spawning through 0.5 mL of KCl injection instead of a higher volume (1–1.5 mL) reported in literature for *P. lividus* (9, 16, 28, 47, and 51), which may lead to mortality (0.67–85%). By using a small volume, no mortality was found in cultured sea urchins, contributing to the success of culturing up to two years, as reported in the present study.

*P. lividus* is known to be a suitable species for long term captivity [9,56]. Spawning performance of cultured sea urchins has been reported for over a year, and it was found to be higher than that occurring in the wild [28]. By performing fertilization assays, we confirmed the good spawning performance of cultured sea urchins after one year. In addition, for the first time, by comparing these data with those from the wild, we also demonstrated that it is feasible to produce fertilized eggs up to a rearing period of more than 2 years. Thus, the fertilization rate from cultured sea urchins exceeded 80%, according to the acceptability criterion established in ecotoxicology [57]. This high fertilization rate confirms the good quality of gametes—essential for bioassay testing—obtained from rearing organisms for up to two years. This period also included summer, when natural populations of *P. lividus* are devoid of gametes, entering the spent or recovery stage. Temperature represents a limiting factor for the gametogenesis of temperate echinoids [10,58,59,60,61] living on the Italian coast. During summer, a high number of adults from the wild were induced to spawn compared to cultured sea urchins. However, in both cases we found similar fertilization success and normal development (Figure 1), confirming that the good quality of gametes of cultured sea urchins is comparable to that sampled in the natural environment during the warmest periods (i.e., July and August). Therefore, we found “ready-to-spawn conditions”, avoiding recurrent field collection of mature specimens, especially when temperature is a limiting factor for *P. lividus* gametogenesis.

Eggs fertilized by cultured sea urchins develop into larvae within 72 h. Normal larval development was observed in bred organisms up to 1 year. After this period, a significant percentage of anomalous development linked with rearing conditions occurred. Seventy-two hours after fertilization, sea urchins develop into four-armed plutei. The early larvae from gametes released by 3-month-old captive sea urchin broodstock fully developed, reaching the planktotrophic pluteus stage [62], differently from those with longer captive periods [28]. In this study, the high normal development rate in larvae from cultured sea urchins up to one year is similar to that from wild ones. No seasonal constraints were reported, since the experiments were performed during both gametogenesis season and out-of-season gametogenesis. The high development success in the larvae from cultured sea urchins may be ascribed to rearing conditions. The latter were selected to mirror the best conditions occurring in the wild, where most of *P. lividus* mature in the field [16,62,63]. The abiotic parameters and the diet selected in this study for up to one year seem to promote not only gamete quality but also normal larval development for environmental toxicology assessment, guaranteeing ‘ready to spawn’ conditions. Yet, these conditions were not suitable to keep sea urchin broodstock in captivity for more than one year, when normal development rates observed were lower than in the wild ones. Further studies addressing different rearing conditions for ensuring proper larval development are needed before proposing periods longer than one year for ecotoxicological surveys. The successful maintenance of sea urchins in captivity should also combine a balanced diet with the needs of the organisms [64]. Thus, since *P. lividus* is one of the main consumers of the seagrass *Posidonia oceanica*, further investigations on long term maintenance of this sea urchin species may integrate the diet selected in this study with the seagrass and macroalgae collected on the same site of sea urchin sampling, which may enhance gonad maturation. Macroalgae and patches of *P. oceanica* may be first introduced in the tank before animals to mimic a natural environment and then supplied as diet. In this regard, all parts of *P. oceanica* are consumed by *P. lividus* [65], which is one of the main consumers of the seagrass [66]. *P. oceanica* has been considered a “preferred” species of *P. lividus* for feeding during spring and summer [67]; thus, its presence may promote ‘ready to spawn’ conditions. Moreover, animal protein—such as fish discards (i.e., fins, skin, and bones)—could be supplied in the diet to improve long term gamete quality, since a feed formulation based on a balanced vegetal/animal ratio can be responsible for gonad enhancement in *P. lividus* reared up to three months [29].

**Figure 2:**
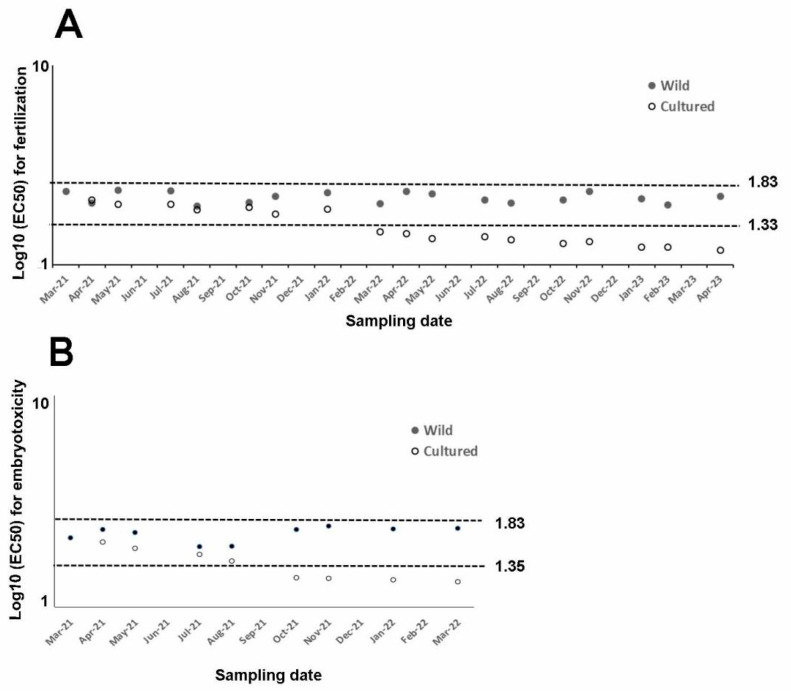
Summary of log10 of EC50 for every assay from wild (filled dots) and cultured sea urchins (empty dots) exposed to copper nitrate and compared to literature figures (dotted lines), (**A**) whose log10 (EC50) varies between fertilization (1.33–1.83) and (**B**) embryotoxicity (1.35–1.83i) assays.

In our case, only sea urchins reared for one year were used to assess embryotoxicity assays, while longer cultured periods (2 years) were selected for fertilization assays. Copper nitrate affected fertilization. Related EC50 from wild or captive sea urchins reported in the literature range between 21.69 µg/L and 68.18 µg/L [43,68,69,70,71,72,73]. They correspond to 1.33 and 1.83 log10 of EC50 (Figure 2A) and are consistent with those reported in this study for both wild and sea urchins cultured for 10 months.

Conversely, toxicity tolerance found in cultured sea urchins since March 2023 lies outside of this range, suggesting that a rearing period longer than 10 months is not suitable to perform fertilization assays using the rearing conditions described in this study.

Regarding embryotoxicity, the EC50 measured in wild sea urchins and cultured sea urchins reared up to August 2021 falls within the range reported in the literature (22.60–68.34 µg/L, corresponding to 1.354 and 1.834 of the log10 [68,73,74,75,76,77,78,79,80], thus demonstrating the good quality of gametes allowing for normal development up to 72 h. Conversely, rearing time of more than five months was not suitable to perform ecotoxicity tests, since reported toxicity would lie out of the literature range.

Overall, biotic and abiotic factors—including pollutants, temperature, pH, food availability and feeding protocols, and photoperiod—may affect sea urchin toxicological endpoints and therefore gamete quality and development. Embryo- and larval toxicity bioassays conducted by exposing several sea urchin species—including *P. lividus*—to metals show different larval sensitivity due to different temperature and diets [14,64]. Likewise, fertilization assay reveals significant differences between wild and captive *P. lividus*, likely due to abiotic factors and diet [51,62]. Accordingly, husbandry conditions affect gamete sensitivity towards contaminants in terms of fertilization rate and larval development, as reported in the present study, when EC50 values were lower than those measured in wild sea urchins. This result may be mainly due to the diet, which should be further improved, for example, by combining vegetables with animal sources, as stated above. However, animal protein percentage should not exceed vegetable content, since a diet with high animal content is responsible for changing organisms’ sensitivity for toxicity tests up to 1 month after rearing [64]. Thus, a nutritionally balanced diet promoting gamete quality is paramount to maintain the reliability of using sea urchin fertilization and development in environmental biomonitoring studies [62].

Sea urchins are promising alternative non-mammalian models for assessing human health risk caused by different contaminants in the marine environment, in line with the Replacement, Reduction, and Refinement (3Rs) principle [81]. According to the 3Rs recommendations, when bioassays target invertebrates, exposures are performed at early developmental stages. Thus, sea urchin fertilization and embryotoxicity assays are used in toxicological studies since they expose the most sensitive stages (gametes and embryos) to pollutants [2,4]. Despite sea urchin toxicity tests having been widely studied and several protocols being available [82], there is an urgent need for standardization to enhance results’ reproducibility in different laboratories, relevance to field conditions, and animal welfare [64]. Although a standardization for *P. lividus* toxicity tests is still lacking, we performed the fertilization and embryotoxicity assays by using the guidelines provided by the Italian Institute for Environmental Protection and Research [42], which has a leading role in standardizing and regulating the use of biological tests in Italian legislation [83].

## 5. Conclusions

This study promotes ‘ready-to-spawn’ conditions for the first time by rearing sea urchins for ecotoxicology surveys, recommending up to 10-month and 5-month rearing times to perform fertilization and embryo toxicity assays, respectively. These findings provide a baseline in marine ecotoxicology to obtain gametes on demand, irrespective of reproductive period and natural or human-driven pressures that may impact gamete availability.

## Figures and Tables

**Table 1:** EC50 values (expressed in µg/L) with 95% confidence limits (C.L.) and converted into logarithmic scale (log10) calculated during fertilization tests (spermiotoxicity) with copper nitrate, from adult sea urchins sampled in the field (wild) and reared in the aquaria (cultured) from March 2021 to April 2023.

Period	EC50 and C.L. Wild	Log10 Wild	EC50 and C.L. Cultured	Log10 Cultured
March 2021	56.46 (48.81–59.02)	1.74	-	-
April 2021	41.18 (35.28–50.61)	1.61	44.25 (40.01–48.84)	1.65
May 2021	55.84 (42.80–59.60)	1.75	40.04 (37.83–44.19)	1.60
July 2021	55.00 (50.02–60.80)	1.74	39.99 (37.84–43.28)	1.60
August 2021	38.75 (34.77–43.18)	1.59	35.27 (33.18–39.24)	1.55
October 2021	41.73 (38.99–44.13)	1.62	37.39 (36.04–38.73)	1.57
November 2021	48.12 (42.09–54.64)	1.68	32.28 (21.18–37.82)	1.51
January 2022	52.41 (50.03–55.28)	1.72	35.75 (31.51–40.55)	1.55
March 2022	40.73 (37.94–44.27)	1.61	21.25 (18.99–23.77)	1.33
April 2022	54.12 (51.19–57.73)	1.73	20.55 (18.73–22.54)	1.31
May 2022	51.04 (49.18–55.29)	1.71	18.11 (16.19–21.02)	1.26
July 2022	44.17 (41.89–48.27)	1.65	18.91 (15.73–21.53)	1.28
August 2022	41.29 (40.04–43.11)	1.62	17.72 (14.18–19.01)	1.25
October 2022	44.73 (39.83–49.58)	1.65	16.19 (13.92–18.99)	1.21
November 2022	54.09 (49.74–58.73)	1.73	16.93 (12.89–17.54)	1.23
January 2023	45.87 (41.25–50.04)	1.66	15.04 (11.97–19.21)	1.18
February 2023	39.43 (36.11–43.09)	1.60	14.87 (13.14–15.95)	1.17
April 2023	48.26 (46.87–51.02)	1.68	13.99 (12.07–16.03)	1.15

**Table 2:** EC50 (expressed in µg/L) with 95% confidence limits (C.L.) and converted into logarithmic scale (log10), calculated during embryotoxicity tests with copper nitrate, from adult sea urchins sampled in the field (wild) and reared in the aquaria (cultured) within one year (March 2021–2022).

Period	EC50 and C.L. Wild	Log10 Wild	EC50 and C.L. Cultured	Log10 Cultured
March 2021	44.94 (31.78–55.72)	1.65	-	-
April 2021	53.85 (49.61–58.46)	1.73	40.99 (38.38–43.28)	1.61
May 2021	50.43 (45.96–55.33)	1.70	35.75 (31.51–49.55)	1.55
July 2021	37.10 (29.53–46.61)	1.57	31.28 (20.17–32.74)	1.50
August 2021	37.40 (36.04–38.73)	1.57	27.11 (25.63–29.51)	1.43
October 2021	53.83 (49.61–58.46)	1.73	20.14 (19.54–21.20)	1.30
November 2021	58.05 (54.98–61.28)	1.76	18.60 (16.81–20.59)	1.27
January 2022	54.46 (48.81–59.06)	1.74	18.01 (17.27–19.83)	1.26
March 2022	55.24 (51.26–59.24)	1.74	17.27 (15.32–19.28)	1.24

## Data Availability

Data supporting reported results can be found within Appendix A in the document.

## Supplementary Materials

The following supporting information can be downloaded at: https://www.mdpi.com/article/10.3390/toxics13080705/s1, Table S1: Parameters—Temperature (T), salinity (S), pH, and dissolved oxygen (DO) measured in the tanks. Figure S1: Images showing sea urchin fertilized eggs (A) and development (B–E). (B) Larva with normal development; anomalous larva with (C) a truncated appearance, (D) crossed tips at the apex, (E) skeletal regression in the arms. Bars equal 100 µm.

## Appendix A

**Table A1:** Ingredients and ratio (%) used to feed adult sea urchins of *P. lividus* cultured in the aquaria. Supplements included fish oil and calcium carbonate.

Ingredient	Ratio (%)
Lettuce	15
Carrots	5
Corns	17
Algae	43
Agar	8
Supplements	12

**Table A2:** Periods—each one corresponding to six months, equivalent to four spawning times—selected for the statistical analysis.

Period (P)	Spawning (Months–Year) Period
P1	April–May–July–August 2021
P2	October–November 2021; January–March 2022
P3	April–May–July–August 2022
P4	October–November 2022; January–February 2023

**Table A3:** Four-month period selected for the statistical analysis. Asterisks indicate significant different values.

Source	Df	SS	MS	Pseudo-F	P(MC)
Status	1	0.13448	0.13488	68.401	0.0001
Period	3	7.41 × 10^−2^	2.47 × 10^−2^	12.526	0.0002
Status × Period	3	5.33 × 10^−2^	1.78 × 10^−2^	9.0123	0.0006 *
Res	24	4.73 × 10^−2^	1.97 × 10^−3^		
Total	31	0.30963			

**Table A4:** Statistical analysis—by using PERMANOVA and the Monte Carlo test—for Experiment I after 72 h from the fertilization in each condition status (wild and cultured conditions) and periods (Period, P). P1: first period; P2: second period; P3: third period; P4: fourth period. Asterisks indicate significant different values.

WILD			CULTURED		
P	*t*	P (MC)	P	*t*	P (MC)
P1–P2	2.0253	0.342	P1–P2	0.55183	0.6059
P3–P4	0.38796	0.7079	P3–P4	5.0482	0.0025 *
P1–P4	0.29001	0.7838	P1–P4	5.7673	0.001 *
P2–P3	1.8191	0.1196	P2–P3	9.164	0.0002 *
P2–P4	1.5421	0.175	P2–P4	7.3644	0.0004 *
P3–P4	0.91696	0.3914	P3–P4	2.6053	0.038 *

**Table A5:** Statistical analysis—by using PERANOVA and the Monte Carlo test—for Experiment I after 72 h from the fertilization by comparing development in larvae obtained from wild and cultured sea urchins of each period (P). Asterisks indicate significant different values.

Groups	*t*	P (MC)
P1–wild cultured	0.657	0.5412
P2–wild cultured	5.0016	0.0028 *
P3–wild cultured	5.2439	0.0023 *
P4–wild cultured	9.3299	0.0002 *

## References

[1] Carreras C., García-Cisneros A., Wangensteen O.S., Ordonez V., Palacin C., Pascual M., Turon X. (2020). East is East and West is West: Population genomics and hierarchical analyses reveal genetic structure and adaptation footprints in the keystone species *Paracentrotus lividus* (Echinoidea). Divers. Distrib..

[2] Beiras R., Fernández N., Bellas J., Besada V., Gonzalez-Quijano A., Nunes T. (2003). Integrative assessment of marine pollution in Galician estuaries using sediment chemistry, mussel bioaccumulation, and embryo-larval toxicity bioassays. Chemosphere.

[3] Pagano G., Guida M., Trifuoggi M., Thomas P., Palumbo A., Romano G., Oral R. (2017). Sea urchin Bioassays in toxicity testing: I. Inorganics, organics, complex mixtures and natural products. Expert Opin. Environ. Biol..

[4] Broccoli A., Morroni L., Valentini A., Vitiello V., Renzi M., Nuccio C., Pellegrini D. (2021). Comparison of different ecotoxicological batteries with WOE approach for the environmental quality evaluation of harbour sediments. Aquat. Toxicol..

[5] Gambardella C., Marcellini F., Falugi C., Varrella S., Corinaldesi C. (2021). Early-stage anomalies in the sea urchin (*Paracentrotus lividus*) as bioindicators of multiple stressors in the marine environment: Overview and future perspectives. Environ. Pollut..

[6] ASTM (1995). Standard Guide for Conducting Static Acute Toxicity Tests with Echinoid Embryos.

[7] ASTM (2004). Standard Guide for Conducting Static Acute Toxicity Tests with Echinoid Embryos.

[8] USEPA Environmental Protection Agency (2002). Method 1008.0: Sea urchin, Arbacia punctulata, Fertilization Test. Chronic Toxicity.

[9] Cirino P., Ciaravolo M., Paglialonga A., Toscano A. (2017). Long-term maintenance of the sea urchin *Paracentrotus lividus* in culture. Aquac. Rep..

[10] Spirlet C., Grosjean P., Jangoux M. (1998). Reproductive cycle of the echinoid *Paracentrotus lividus*: Analysis by means of the maturity index. Invertebr. Reprod. Dev..

[11] Spirlet C., Grosjean P., Jangoux M. (2000). Optimisation of gonad growth by manipulation of temperature and photoperiod in cultivated sea urchins, *Paracentrotus lividus* (Lamarck) (Echinodermata). Aquaculture.

[12] Byrne M., Ho M., Selvakumaraswamy P., Nguyen H.D., Dworjanyn S.A., Davis A.R. (2009). Temperature, but not pH, compromises sea urchin fertilization and early development under near-future climate change scenarios. Proc. R. Soc. B Biol. Sci..

[13] Sartori D., Lera S., Giuliani S., Macchia S., Morroni L., Pellegrini D., Gaion A., Agnello M. (2017). Importance of Gamete Quality in Ecotoxicological Application: Natural versus Bred Population in *Paracentrotus lividus*. Sea Urchin—From Environment to Aquaculture and Biomedicine.

[14] Sartori D., Scatena C., Vrinceanu C.A., Gaion A. (2023). Increased sensitivity of sea urchin larvae to metal toxicity as a consequence of the past two decades of climate change and ocean acidification in the Mediterranean Sea. Mar. Pollut. Bull..

[15] Toso A., Necci F., Martines A., Lacorte R., Toso Y., Gianguzza P., Deidun A., Ungaro N., Costantino G., Caforio M. (2025). Overfishing and sea warming drive the collapse of *Paracentrotus lividus*. Sci. Rep..

[16] Gago J., Luis O.J. (2011). Comparison of spawning induction techniques on *Paracentrotus lividus* (Echinodermata: Echinoidea) broodstock. Aquacult. Int..

[17] Carboni S., Vignier J., Chiantore M.C., Tocher D.R., Migaud H. (2012). Effects of dietary microalgae on growth, survival and fatty acid composition of sea urchin *Paracentrotus lividus* throughout larval development. Aquaculture.

[18] Carboni S., Hughes A.D., Atack T., Tocher D.R., Migaud H. (2013). Influence of broodstock diet on somatic growth, fecundity, gonad carotenoids and larval survival of sea urchin. Aquac. Res..

[19] Carboni S., Kelly M.S., Hughes A.D., Vignier J., Atack T., Migaud H. (2014). Evaluation of flow through culture technique for commercial production of sea urchin (*Paracentrotus lividus*) larvae. Aquac. Res..

[20] De La Uz S., Carrasco J.F., Rodrıguez C., Anadon N. (2013). Metamorphosis, growth and survival of early juveniles of *Paracentrotus lividus* (Echinodermata: Echinoidea): Effects of larval diet and settlement inducers. Cah. De Biol. Mar..

[21] Paredes E., Bellas J., Costas D. (2015). Sea urchin (*Paracentrotus lividus*) larval rearing—Culture from cryopreserved embryos. Aquaculture.

[22] Brundu G., Vallaine D., Baroli M., Figus A.M., Pinna A., Carboni S. (2016). Effects of on-demand feeding on sea urchin larvae (*Paracentrotus lividus*; Lamarck, 1816), development, survival and microalgae utilization. Aquac. Res..

[23] Gharbi M., Glaviano F., Federico S., Pinto B., Cosmo A.D., Costantini M., Zupo V. (2023). Scale-up of an aquaculture plant for reproduction and conservation of the sea urchin *Paracentrotus lividus*: Development of post-larval feeds. J. Mar. Sci. Eng..

[24] Shpigel M., McBride S.C., Marciano S., Lupatsch I. (2004). The effect of photoperiod and temperature on the reproduction of European sea urchin *Paracentrotus lividus*. Aquaculture.

[25] Santos P.M., Albano P., Raposo A., Ferreira S.M.F., Costa J.L., Pombo A. (2020). The effect of temperature on somatic and gonadal development of the sea urchin *Paracentrotus lividus* (Lamarck, 1816). Aquaculture.

[26] Bennett J., Mos B., Dworjanyn S.A. (2024). Shipping live sea urchins: Effects of temperature and exposure time on survival. Aquaculture.

[27] Ruocco N., Zupo V., Caramiello D., Glaviano F., Polese G., Albarano L., Costantini M. (2018). Experimental evaluation of the feeding rate, growth and fertility of the sea urchins *Paracentrotus lividus*. Invertebr. Reprod. Dev..

[28] Luis O., Delgado F., Gago J. (2005). Year-round captive spawning performance of the sea urchin *Paracentrotus lividus*: Relevance for the use of its larvae as live feed. Aquat. Living Resour..

[29] Ciriminna L., Signa G., Vaccaro A.M., Visconti G., Mazzola A., Vizzini S. (2021). Turning waste into gold: Sustainable feed made of discards from the food industries promotes gonad development and colouration in the commercial sea urchin *Paracentrotus lividus* (Lamarck, 1816). Aquac. Rep..

[30] Ciriminna L., Rakay A., Grosso L., Pensa D., Fianchini A., Mazzola A., Vizzini S. (2024). Evaluation of sustainable feeds for “caviar” production in the Mediterranean sea urchin *Paracentrotus lividus* (Lamarck, 1816). Aquac. Rep..

[31] Dupont S., Thorndyke M.C. (2009). Impact of CO_2_-driven ocean acidification on invertebrates early life-history—What we know, what we need to know and what we can do. Biogeosci. Discuss..

[32] Pagano G., Romana L. (1991). L’utilisation des oursins comme témoins de contamination. Tests biologiques sur les embryons et le sperme des oursins pour la surveillance de la pollution marine. Océanis.

[33] Bellas J., Fernandez N., Lorenzo I., Beiras R. (2008). Integrative assessment of coastal pollution in a Ria coastal system (Galicia, NW Spain): Correspondence between sediment chemistry and toxicity. Chemosphere.

[34] Pétinay S., Chataigner C., Basuyaux B. (2009). Standardisation du développement larvaire de l’oursin, *Paracentrotus lividus*, pour l’évaluation de la qualité d’une eau de mer. C. R. Biol..

[35] Morroni L., Sartori D., Costantini M., Genovesi L., Magliocco T., Ruocco N., Buttin I. (2019). First molecular evidence of the toxicogenetic effects of copper on sea urchin *Paracentrotus lividus* embryo development. Water Res..

[36] Gambardella C., Miroglio R., Prieto Amador M., Castelli F., Castellano L., Piazza V., Faimali M., Garaventa F. (2024). High concentrations of phthalates affect the early development of the sea urchin *Paracentrotus lividus*. Ecotox. Environ. Saf..

[37] US EPA (1994). Sea Urchin, *Arbacia punctulata*, Fertilization Test Method 1008.0. Short-Term Methods for Estimating the Chronic Toxicity of Effluents and Receiving Waters to Marine and Estuarine Organisms.

[38] US EPA (1995). Purple Sea Urchin, *Strongylocentrotus purpuratus* and Sand Dollar, *Dendraster excentricus* Fertilization Test Method. Short-Term Methods for Estimating the Chronic Toxicity of Effluents and Receiving Waters to West Coast Marine and Estuarine Organisms.

[39] OECD (1998). OECD Series on Testing and Assessment Number 11 Detailed Review Paper on Aquatic Testing Methods for Pesticides and Industrial Chemicals.

[40] Environmental Canada (1992). Biological Test Method: Fertilization Assay Using Echinoids (Sea Urchins and Sand Dollars).

[41] ICES (2003). Report of the ICES Advisory Committee on the Marine Environment.

[42] ISPRA (2017). Saggio di Fecondazione e Saggio di Sviluppo Embrionale con il Riccio di Mare Paracentrotus lividus (Lamarck) (Echinodermata: Echinoidea). Quaderni di Ecotossicologia.

[43] His E., Heyvang I., Geffard O., Montaudouin X. (1999). A comparison between oyster (*Crassostrea gigas*) and sea urchin (*Paracentrotus lividus*) larval bioassays for toxicological studies. Water Res..

[44] Chapman G.A. (1995). Sea urchin sperm cell test. Fundamentals of Aquatic Toxicology: Effects, Environmental Fate and Risk Assessment.

[45] Cyrus M.D., Bolton J.J., Scholtz R., Macey B.M. (2014). The advantages of *Ulva* (Chlorophyta) as an additive in sea urchin formulated feeds: Effects on palatability, consumption and digestibility. Aquac. Nutr..

[46] Cyrus M.D., Bolton J.J., De Wet L., Macey B.M. (2015). The role of the green seaweed *Ulva* as a dietary supplement for full life-cycle grow-out of *Tripneustes gratilla*. Aquaculture.

[47] Raposo A., Ferreira S.M., Ramos R., Anjos C.M., Baptista T., Tecelao C., Goncavales S.C., Pombo A. (2016). Effect of three diets in the gonadal growth and maturation of Paracentrotus lividus. Frontiers in Marine Science Conference Abstract: IMMR | International Meeting on Marine Research Peniche, Portugal.

[48] Sartori D., Pellegrini D., Macchia S., Gaion A. (2016). Can echinoculture be a feasible and effective activity? Analysis of fast reliable breeding conditions to promote gonadal growth and sexual maturation in *Paracentrotus lividus*. Aquaculture.

[49] Rakay A., Grosso L., Fianchini A., Cataudella S. (2024). Raking. A sustainable no-kill sea urchin aquaculture method to obtain caviar. Nat. Sustain..

[50] Gambardella C., Miroglio R., Trenti F., Guella G., Sbrana F., Grunder M., Garaventa F., Sepcic K. (2023). Assessing the toxicity of aegerolysin-based bioinsecticidal complexes using the sea urchin *Paracentrotus lividus* as model organism. Aquat. Toxicol..

[51] Luis R., Josè R., Castro J., Andrade G. (2023). Advances in aquaculture hatchery techniques of sea urchin *Sphaerechinus granularis* (Lamarck, 1816) (Echinoidea: Toxopneustidae): Broodstock conditioning and spawning induction. Life.

[52] Asnicar D., Locatello L., Zanovello L., Minichino R., Masiero L., Munari M., Marin M.G. (2024). How do sea urchins prepare offspring to face ocean acidification? Gamete intraspecific differences and adaptability. Front. Mar. Sci..

[53] Manzo S. (2004). Sea urchin embryotoxicity test: Proposal for a simplified bioassay. Ecotoxicol. Environ. Saf..

[54] Volpi Ghirardini A., Arizzi Novelli A., Tagliapietra D. (2005). Sediment toxicity assessment in the Lagoon of Venice (Italy) using *Paracentrotus lividus* (Echinodermata: Echinoidea) fertilization and embryo bioassays. Environ. Int..

[55] Gambardella C., Aluigi M.G., Ferrando S., Gallus L., Ramoino P., Gatti A.M., Rottigni M., Falugi C. (2013). Developmental abnormalities and changes in cholinesterase activity in sea urchin embryos and larvae from sperm exposed to engineered nanoparticles. Aquat. Toxicol..

[56] Brundu G., Farina S., Domenici P. (2020). Going back into the wild: The behavioural effects of raising sea urchins in captivity. Conserv. Physiol..

[57] US EPA, Environmental Protection Agency (2000). Method Guidance and Recommendations for Whole Effluent Toxicity (WET) Testing (40 CFR Part 136).

[58] Byrne M. (1990). Annual reproductive cycles of the commercial sea urchin *Paracentrotus lividus* from an exposed intertidal and a sheltered subtidal habitat on the west coast of Ireland. Mar. Biol..

[59] Lozano J., Galera J., Lopez S., Turon X., Palacin C., Morera G. (1995). Biological cycles and recruitment of *Paracentrotus lividus* (Echinodermata: Echinoidea) in two contrasting habitats. Mar. Ecol. Progr. Ser..

[60] Guettaf M. (1997). Contribution à l’étude de la variabilité du cycle reproducteur (indice gonadique et histologie des gonades) chez *Paracentrotus lividus* (Echinodermata: Echinoidea) en Méditerranée sud occidentale (Algérie). Ph.D. Thesis.

[61] Martinez I., Garcia F.J., Sanchez A.I., Daza J.L., del Castillo F., Feral J.F., David B. (2003). Biometric Parameters and Reproductive Cycle of Paracentrotus Lividus (Lamarck) in Three Habitats of Southern Spain.

[62] De Rosa Repolho T.F.P., Costa M.H., de Jesus Luis O., de Matos Gago J.A.E. (2011). Broodstock diet effect on sea urchin *Paracentrotus lividus* (Lamarck, 1816) endotrophic larvae development: Potential for their year-round use in environmental toxicology assessment. Environ. Ecotoxicol. Saf..

[63] Gago J., Range P., Luıs O.J., Feral J.F., David B. (2003). Growth, Reproductive Biology and Habitat Selection of the Sea Urchin Paracentrotus Lividus in the Coastal Waters of Cascais, Portugal.

[64] Soares J.B., Resgalla C.J. (2016). Echinodermata in ecotoxicological tests: Maintenance and sensitivity. Braz. J. Oceanogr..

[65] Tejada S., Deudero S., Box A., Sureda A. (2013). Physiological response of the sea urchin *Paracentrotus lividus* fed with the sea grass *Posidonia oceanica* and the alien algae *Caulerpa racemosa* and *Lophocladia lallemandii*. Mar. Environ. Res..

[66] Verlaque M. (1987). Relations Entre Paracentrotus lividus (Lmk) Et Le Phytobenthos de Méditerrané Occidentale, Boudouresque, C.F., Colloque Internationale sur Paracentrotus lividus Et Les Oursins Comestibles.

[67] Boudouresque C.F., Verlaque M., Lawrence J.M. (2001). Ecology of *Paracentrotus lividus*. Edible Sea Urchins: Biology and Ecology.

[68] Dinnel P.A., Link J.M., Stober Q.J. (1987). Improved methodology for a sea urchin sperm cell bioassay for marine waters. Arch. Environ. Contam. Toxicol..

[69] George S.B., Lawrence J.M., Lawrence A.L., Ford J. (2000). Fertilization and development of the sea urchin *Lytechinus variegatus* maintained on an extruded feed. J. World Aquac. Soc..

[70] Radenac G., Fichet D., Miramand P. (2001). Bioaccumulation and toxicity of four dissolved metals in *Paracentrotus lividus* sea-urchin embryo. Mar. Environ. Res..

[71] Volpi Ghirardini A., Arizzi Novelli A. (2001). A sperm cell toxicity test procedure for the Mediterranean species *Paracentrotus lividus* (Echinodermata: Echinoidea). Environ. Technol..

[72] Beiras R., Bellas J., Fernandez N., Lorenzo J.I., Garcıa A.C. (2003). Assessment of coastal marine pollution in Galicia (NW Iberian Peninsula): Metal concentrations in seawater, sediments and mussels (*Mytilus galloprovincialis*) versus embryo-larval bioassays using *Paracentrotus lividus* and *Ciona intestinalis*. Mar. Environ. Res..

[73] Lera S., Pellegrini D. (2006). Evaluation of the fertilization capability of *Paracentrotus lividus* sea urchin storaged gametes by the exposure to different aqueous matrices. Environ. Monit. Assess..

[74] Nacci D., Jackim E., Walsh R. (1986). Comparative evaluation of three rapid marine toxicity tests: Sea urchin early embryo growth test, sea urchin sperm cell toxicity test and microtox. Environ. Toxicol. Chem..

[75] Pagano G., Anselmi B., Dinnel P.A., Esposito A., Guida M., Iaccarino M., Melluso G., Pascale M., Trieff N.M. (1993). Effects on sea urchin fertilization and embryogenesis of water and sediment from two rivers in Campania, Italy. Bull. Environ. Contam. Toxicol..

[76] Pagano G., Korkina L.G., Iaccarino M., De Biase A., Deeva I.B., Doronin Y.K., Guida M., Melluso G., Meriç S., Oral R., Garrigues P., Walker C.H., Barth H. (2001). Developmental, cytogenetic and biochemical effects of spiked or environmentally polluted sediments in sea urchin bioassays. Biomarkers in Marine Ecosystems: A Practical Approach.

[77] Fernández N., Beiras R. (2001). Combined toxicity of dissolved mercury with copper, lead and cadmium on embryogenesis and early larval growth of the *Paracentrotus lividus* sea urchin. Ecotoxicology.

[78] Anderson B., Nicely P., Gilbert K., Kosaka R., Hunt J., Phillips B. (2004). Overview of Freshwater and Marine Toxicity Tests: A Technical Tool for Ecological Risk Assessment.

[79] Arizzi Novelli A., Losso C., Ghetti P.F., Volpi Ghirardini A. (2003). Toxicity of heavy metal using sperm cell and embryo toxicity with *Paracentrotus lividus* (Echinodermata: Echinoidea): Comparison with exposure concentration in the lagoon of Venice, Italy. Environ. Toxicol. Chem..

[80] Marin M.G., Da Los L., Moschino V., Campesan G. (2001). Sediment elutriate toxicity testing with embryos of sea urchin (*Paracentrotus lividus*). Aquat. Ecosyst. Health Manag..

[81] Pinsino A., Alijagic A. (2019). Sea urchin *Paracentrotus lividus* immune cells in culture: Formulation of the appropriate harvesting and culture media and maintenance conditions. Biol. Open.

[82] Buric P., Kovacic I., Ilic K., Winter D.S., Bursic M. (2025). A decade of toxicity research on sea urchins: A review. Toxicon.

[83] ICRAM (2007). Manuale Per La Movimentazione Di Sedimenti Marini. https://www.isprambiente.gov.it/contentfiles/00006700/6770-manuale-apat-icram-2007.pdf/.

