# Supplementary material for: Rearing Sea Urchins to Promote ‘Ready-to-Spawn’ Conditions for Ecotoxicological Surveys
Source: Toxics. 2025 Aug 21;13(8):705. doi: 10.3390/toxics13080705 (PMC12389862; doi:10.3390/toxics13080705)

**Supplementary File**

**Supplementary Table.** Temperature (T), salinity (S), pH and dissolved oxygen (DO) measured in the tanks.

| Month | Week | Temperature(℃) | Salinity (‰) | pH | DO (%) |
|---|---|---|---|---|---|
| March 2021 | 1 week | 16.5 | 35.1 | 8.00 | 94.3 |
| | 2 week | 16.5 | 35.2 | 8.01 | 90.4 |
| | 3 week | 16.0 | 35.1 | 8.05 | 93.2 |
| | 4 week | 16.5 | 35.5 | 8.10 | 95.0 |
| April 2021 | 1 week | 16.0 | 36.0 | 8.04 | 90.5 |
| | 2 week | 16.3 | 36.0 | 8.00 | 93.2 |
| | 3 week | 16.2 | 36.5 | 8.00 | 95.8 |
| | 4 week | 16.0 | 36.0 | 8.05 | 92.8 |
| May 2021 | 1 week | 16.6 | 36.5 | 8.10 | 95.5 |
| | 2 week | 16.0 | 35.3 | 8.05 | 95.7 |
| | 3 week | 16.5 | 35.5 | 8.12 | 93.5 |
| | 4 week | 16.6 | 36.0 | 8.20 | 97.3 |
| Juny 2021 | 1 week | 17.0 | 37.0 | 8.15 | 96.6 |
| | 2 week | 16.4 | 36.5 | 8.10 | 95.4 |
| | 3 week | 16.5 | 36.0 | 8.11 | 96.2 |
| | 4 week | 16.5 | 35.4 | 8.05 | 93.8 |
| July 2021 | 1 week | 17.0 | 35.0 | 8.15 | 94.7 |
| | 2 week | 16.8 | 35.0 | 8.20 | 96.5 |
| | 3 week | 16.5 | 35.5 | 8.20 | 95.3 |
| | 4 week | 16.5 | 36.0 | 8.13 | 96.8 |
| August 2021 | 1 week | 16.0 | 36.5 | 8.1 | 97.0 |
| | 2 week | 16.0 | 37.0 | 8.06 | 95.0 |
| | 3 week | 16.5 | 36.5 | 8.10 | 94.3 |
| | 4 week | 17.0 | 35.6 | 8.10 | 95.5 |
| September 2021 | 1 week | 17.0 | 35.5 | 8.10 | 92.5 |
| | 2 week | 16.4 | 35.0 | 8.15 | 93.0 |
| | 3 week | 16.5 | 35.0 | 8.05 | 95.1 |
| | 4 week | 16.0 | 35.5 | 8.10 | 96.2 |
| October 2021 | 1 week | 16.0 | 36.0 | 8.10 | 94.6 |
| | 2 week | 16.0 | 36.2 | 8.15 | 95.6 |
| | 3 week | 16.5 | 36.0 | 8.05 | 97.3 |
| | 4 week | 17.0 | 35.5 | 8.06 | 96.0 |
| November 2021 | 1 week | 16.8 | 35.0 | 8.07 | 95.1 |
| | 2 week | 16.5 | 35.5 | 8.10 | 96.1 |
| | 3 week | 16.4 | 36.0 | 8.11 | 94.3 |
| | 4 week | 17.0 | 35.5 | 8.06 | 93.2 |
| December 2021 | 1 week | 16.0 | 35.5 | 8.00 | 95.1 |
| | 2 week | 16.3 | 35.0 | 8.10 | 94.5 |
| | 3 week | 16.5 | 36.2 | 8.00 | 93.7 |
| | 4 week | 16.0 | 36.5 | 8.15 | 95.0 |
| January 2022 | 1 week | 17.0 | 37.0 | 8.10 | 94.2 |
| | 2 week | 16.5 | 36.5 | 8.10 | 94.7 |

| | | | | | |
|---|---|---|---|---|---|
| | 3 week | 16.0 | 36.0 | 8.10 | 96.5 |
| | 4 week | 17.0 | 36.0 | 8.07 | 96.1 |
| February 2022 | 1 week | 16.5 | 35.8 | 8.00 | 95.1 |
| | 2 week | 16.4 | 35.0 | 8.10 | 95.4 |
| | 3 week | 16.0 | 35.1 | 8.15 | 93.2 |
| | 4 week | 16.0 | 35.5 | 8.12 | 94.6 |
| March 2022 | 1 week | 16.3 | 36.0 | 8.13 | 95.2 |
| | 2 week | 16.5 | 36.3 | 8.15 | 97.0 |
| | 3 week | 17.0 | 36.4 | 8.15 | 94.6 |
| | 4 week | 16.0 | 36.0 | 8.15 | 94.6 |
| April 2022 | 1 week | 16.5 | 35.0 | 8.00 | 95.1 |
| | 2 week | 16.4 | 35.4 | 8.05 | 96.2 |
| | 3 week | 16.0 | 35.0 | 8.06 | 96.1 |
| | 4 week | 16.0 | 35.5 | 8.10 | 97.4 |
| May 2022 | 1 week | 16.3 | 36.0 | 8.16 | 95.4 |
| | 2 week | 16.5 | 36.0 | 8.20 | 95.5 |
| | 3 week | 16.5 | 36.7 | 8.16 | 96.1 |
| | 4 week | 17.0 | 36.0 | 8.07 | 94.8 |
| Juny 2022 | 1 week | 16.3 | 35.8 | 8.08 | 95.3 |
| | 2 week | 16.5 | 35.5 | 8.10 | 95.2 |
| | 3 week | 16.5 | 35.0 | 8.10 | 96.1 |
| | 4 week | 16.5 | 36.0 | 8.11 | 95.6 |
| July 2022 | 1 week | 16.4 | 36.0 | 8.14 | 96.4 |
| | 2 week | 17.0 | 35.5 | 8.12 | 96.8 |
| | 3 week | 16.0 | 35.3 | 8.00 | 96.1 |
| | 4 week | 16.5 | 36.0 | 8.05 | 98.0 |
| August 2022 | 1 week | 16.5 | 36.5 | 8.13 | 96.3 |
| | 2 week | 17.0 | 36.0 | 8.10 | 96.6 |
| | 3 week | 17.0 | 36.0 | 8.06 | 96.0 |
| | 4 week | 17.0 | 36.6 | 8.17 | 95.8 |
| September 2022 | 1 week | 16.5 | 35.5 | 8.20 | 94.5 |
| | 2 week | 16.5 | 35.1 | 8.18 | 95.3 |
| | 3 week | 16.0 | 35.6 | 8.15 | 95.1 |
| | 4 week | 16.0 | 35.5 | 8.08 | 93.6 |
| October 2022 | 1 week | 16.3 | 36.3 | 8.10 | 92.5 |
| | 2 week | 16.0 | 36.5 | 8.14 | 94.1 |
| | 3 week | 16.0 | 37.0 | 8.10 | 95.1 |
| | 4 week | 16.5 | 36.5 | 8.08 | 95.4 |
| November 2022 | 1 week | 17.0 | 36.0 | 8.03 | 96.3 |
| | 2 week | 16.5 | 35.7 | 8.07 | 96.3 |
| | 3 week | 16.3 | 36.0 | 8.10 | 95.3 |
| | 4 week | 16.0 | 35.0 | 8.06 | 97.1 |
| December 2022 | 1 week | 17.0 | 35.5 | 8.10 | 96.2 |
| | 2 week | 16.8 | 36.0 | 8.13 | 95.8 |
| | 3 week | 16.5 | 35.5 | 8.10 | 96.2 |
| | 4 week | 16.6 | 36.0 | 8.11 | 93.7 |
| January 2023 | 1 week | 16.3 | 36.0 | 8.07 | 94.3 |
| | 2 week | 17.0 | 35.5 | 8.00 | 93.8 |
| | 3 week | 16.6 | 35.0 | 8.08 | 91.8 |
| | 4 week | 16.8 | 36.0 | 8.13 | 92.6 |
| February 2023 | 1 week | 16.5 | 36.6 | 8.14 | 95.2 |
| | 2 week | 17.0 | 36.7 | 8.12 | 95.3 |

| | | | | | |
|---|---|---|---|---|---|
| | 3 week | 17.0 | 36.0 | 8.13 | 96.1 |
| | 4 week | 16.0 | 36.5 | 8.15 | 94.3 |
| March 2023 | 1 week | 16.0 | 35.4 | 8.17 | 93.6 |
| | 2 week | 16.5 | 35.2 | 8.10 | 94.2 |
| | 3 week | 16.2 | 35.0 | 8.06 | 92.4 |
| | 4 week | 16.0 | 36.0 | 8.04 | 95.2 |
| April 2023 | 1 week | 16.0 | 36.6 | 8.08 | 96.3 |

**Supplementary Figure S1.** Images showing sea urchin fertilized eggs (A) and development (B-E). B) Larva with normal development; anomalous larva with C) a truncated appearance, D) crossed tips at the apex, E) skeletal regression in the arms. Bars equal 100 μm.

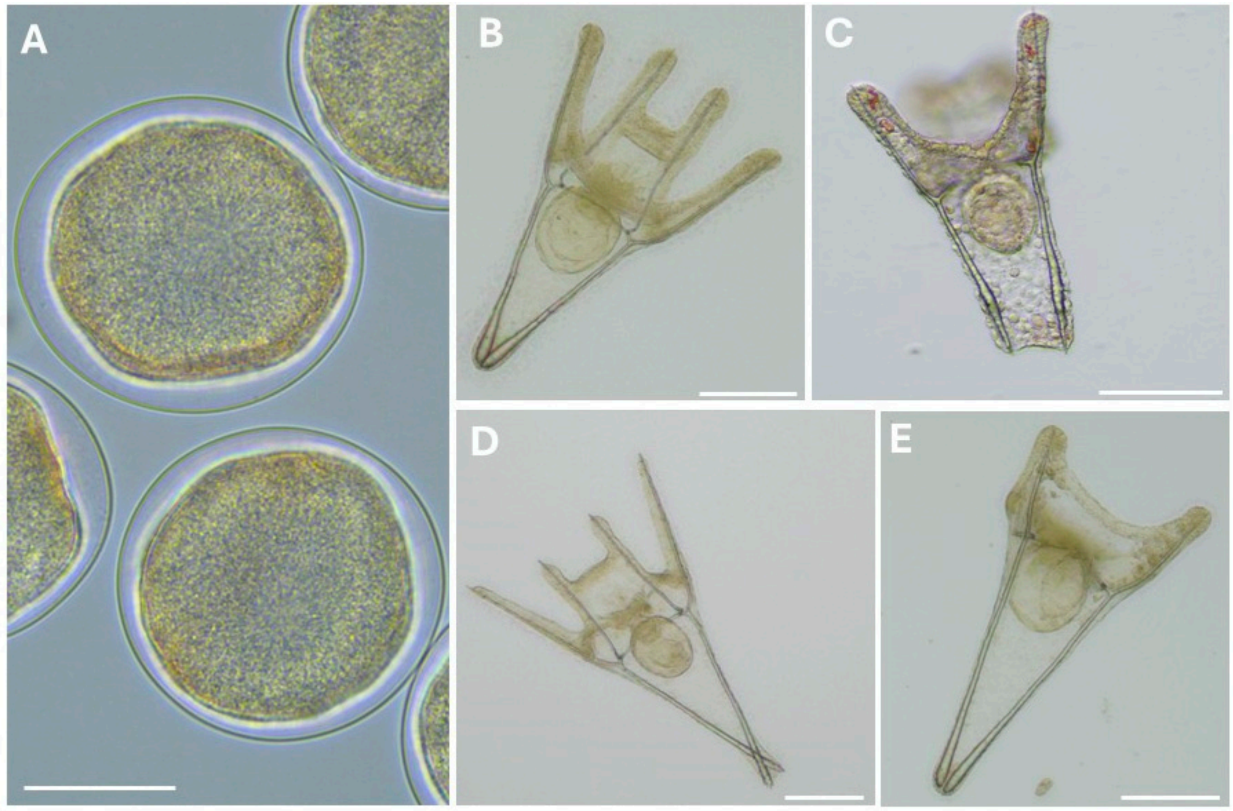

Supplement: Supplementary file 1 [file toxics-13-00705-s001.zip › toxics-3802501-supplementary.pdf]
